# The Clinical Value of GDF15 and Its Prospective Mechanism in Sepsis

**DOI:** 10.3389/fimmu.2021.710977

**Published:** 2021-09-08

**Authors:** Huan Li, Dongling Tang, Juanjuan Chen, Yuanhui Hu, Xin Cai, Pingan Zhang

**Affiliations:** Department of Clinical Laboratory, Renmin Hospital of Wuhan University, Wuhan, China

**Keywords:** sepsis, GDF15, inflammation, JAK1/STAT3, NF-κB p65, biomarker, macrophage polarization, phagocytosis and sterilization

## Abstract

Growth differentiation factor 15 (GDF15) is involved in the occurrence and development of many diseases, and there are few studies on its relationship with sepsis. This article aims to explore the clinical value of GDF15 in sepsis and to preliminarily explore its prospective regulatory effect on macrophage inflammation and its functions. We recruited 320 subjects (132 cases in sepsis group, 93 cases in nonsepsis group, and 95 cases in control group), then detected the serum GDF15 levels and laboratory indicators, and further investigated the correlation between GDF15 and laboratory indicators, and also analyzed the clinical value of GDF15 in sepsis diagnosis, severity assessment, and prognosis. *In vitro*, we used LPS to stimulate THP-1 and RAW264.7 cells to establish the inflammatory model, and detected the expression of GDF15 in the culture medium and cells under the inflammatory state. After that, we added GDF15 recombinant protein (rGDF15) pretreatment to explore its prospective regulatory effect on macrophage inflammation and its functions. The results showed that the serum GDF15 levels were significantly increased in the sepsis group, which was correlated with laboratory indexes of organ damage, coagulation indexes, inflammatory factors, and SOFA score. GDF15 also has a high AUC in the diagnosis of sepsis, which can be further improved by combining with other indicators. The dynamic monitoring of GDF15 levels can play an important role in the judgment and prognosis of sepsis. In the inflammatory state, the expression of intracellular and extracellular GDF15 increased. GDF15 can reduce the levels of cytokines, inhibit M1 polarization induced by LPS, and promote M2 polarization. Moreover, GDF15 also enhances the phagocytosis and bactericidal function of macrophages. Finally, we observed a decreased level of the phosphorylation of JAK1/STAT3 signaling pathway and the nuclear translocation of NF-κB p65 with the pretreatment of rGDF15. In summary, our study found that GDF15 has good clinical application value in sepsis and plays a protective role in the development of sepsis by regulating the functions of macrophages and inhibiting the activation of JAK1/STAT3 pathway and nuclear translocation of NF-κB p65.

## Introduction

Sepsis is a life-threatening organ dysfunction caused by a dysregulated host response to infection, which focuses on the disordered inflammatory response and organ damage in the course of sepsis (1). Due to the high mortality of sepsis (2, 3), its early warning is of great significance to improve the survival rate and prognosis. Although procalcitonin (PCT) is the most widely used sepsis-related biomarker (4), it also may increase in some patients with autoimmune diseases (5) and the level may remain unchanged when virus infection occurs (6). Therefore, it needs to be combined with other laboratory indicators to improve the diagnosis and prognosis value of sepsis.

Growth differentiation factor 15 (GDF15) is a member of the transforming growth factor-β (TGF-β) superfamily. Under physiological conditions, it is generally only expressed in the placenta and prostate, but in pathological conditions such as inflammation, the expression of GDF15 in the tissues and blood is significantly increased (7). Like other members of the TGF-β superfamily, the effect of GDF15 depends on the cellular environment, the stage of the disease, and the microenvironment. At present, many studies have reported that GDF15 is upregulated in many disease processes such as heart failure (8, 9), myocardial infarction (10), pulmonary embolism (11), liver injury (12), and so on. It may be used as a biomarker for these diseases in the future. However, it is unclear whether its upregulation is due to its protective effect or further promotion of disease progression. There were studies reported that GDF15 was elevated in sepsis (13–17), and Fujita et al. reported that the increased GDF15 was a result of mitochondrial stress in sepsis (18).

In terms of the mechanisms, the normal inflammatory response will be out of control when sepsis occurs. A waterfall effect will form through positive feedback, which in turn cause the release of toxic substances and destroy the functions of cells and tissues (19–21). Macrophages play an important role in the inflammatory response of sepsis; they are activated to express a large number of pro-inflammatory factors and further promote the inflammatory response of sepsis (22). Meanwhile, the phagocytosis and sterilization of macrophages could not be ignored when pathogens invaded, while the functions were inhibited in sepsis (23). Therefore, it is particularly important to improve the phagocytosis of macrophages while controlling inflammation in the treatment of sepsis. Several literatures concluded that the increase of GDF15 could protect organ damage in sepsis (24, 25), and some researchers proposed that GDF15 played a protective role in sepsis by improving the tolerance to inflammation (17, 26). However, Santos et al. found that the inhibition of GDF15 could improve the body ability to eliminate pathogens (27), which was inconsistence with the above studies. Taken together, it seems more inclined to consider that GDF15 is involved in the progression of sepsis as a protective factor at present. In the present study, we suspect that GDF15 may regulate the occurrence and development of sepsis through macrophages. Our study found that GDF15 had high clinical value in sepsis, and its dynamic monitoring could indicate the direction of the disease. At the same time, GDF15 could alleviate the inflammatory response by regulating the function of macrophages and reducing the phosphorylation of JAK1/STAT3 and the nuclear translocation of NF-κB p65, which confirmed that GDF15 played a protective role in sepsis from the cellular level.

## Materials and Methods

### Study Population

This study was approved by the Ethics Committee of the Renmin Hospital of Wuhan University (No. WDRY2020-K223), and confirmed to exempt patients from informed consent. All subjects were recruited from the Renmin Hospital of Wuhan University from July 2020 to March 2021. A total of 320 subjects were included, including 132 cases in sepsis group (age: 56.88 ± 14.21 years), 93 cases in nonsepsis group (age: 56.10 ± 14.96 years), and 95 cases in control group (age: 56.00 ± 13.94 years). The inclusion criteria of sepsis group were SOFA ≥ 2 with definite infection. Referring to the Third Edition of Sepsis Expert Consensus (1), sepsis patients were divided into sepsis group and septic shock group, and the specific standards are as following: Patients with septic shock can be identified with a clinical construct of sepsis with persisting hypotension requiring vasopressors to maintain MAP ≥ 65 mmHg and having a serum lactate level > 2 mmol/L (18 mg/dl) despite adequate volume resuscitation. Meanwhile, we divided the patients with sepsis into survival group and death group according to their survival status at 28 days after admission. Patients under 18 years old, pregnant, complicated with tumor and organ function impairment caused by non-infection were excluded. The nonsepsis group is the patients with inflammatory infection but not diagnosed as sepsis, and the exclusion criteria are the same as those in the sepsis group. The control group was the subjects who came to hospital for physical examination during the same period, and excluded subjects who were under 18 years old, accompanied by organ function impairment, had signs of infection in the previous month, or had received antibacterial drugs or other non-preventive drugs for other reasons.

### Sample Preparation

Blood samples were obtained in the morning after overnight fasting within 24 h after hospital admission. Venous blood samples were collected into tubes and centrifuged at 3500 rpm/min for 15 min at 25°C. Plasma and serum were separated and stored at −80°C until analysis.

### Laboratory Analyses

The enzyme-linked immunosorbent assay (ELISA) kit (catalog number: DY957, DY008, R&D Systems, USA) was used to detect serum GDF15 levels. The reference detection range of the kit is 0~0.5 ng/ml, and the sample that exceeded the detection range was diluted and retested. The cobas 8000 e801 automatic chemiluminescence immunoassay analyzer produced by Roche Diagnostics was used to detect serum PCT levels. The red blood cell (RBC), white blood cell (WBC), platelet (PLT) count, and hemoglobin (Hb) levels were detected by the Sysmex XE-2100 automatic blood cell analyzer, and the C-reactive protein (CRP) and serum amyloid A (SAA) were detected by the automatic protein analyzer H780-3 produced by Shenzhen Xilaiheng Company. Serum aspartate aminotransferase (AST), alanine aminotransferase (ALT), glutamyl transferase (GGT), total bilirubin (TBIL), direct bilirubin (DBIL), urea (Urea), creatinine (Crea), and uric acid (UA) used Siemens ADVIA 2400 biochemical analyzer and related reagents to detect. N-terminal pro-brain natriuretic peptide (NT-proBNP), cardiac troponin (cTnI), and creatine kinase-MB (CK-MB) were detected by the Siemens Luminescence Immunoassay Analyzer Centaur XP. Activated partial thromboplastin time (APTT), prothrombin time (PT), thrombin time (TT), and fibrinogen (FIB) were detected by CA7000 automatic coagulation analyzer manufactured by Sysmex, Japan. Interleukin 6 (IL-6) was detected by the cyclic enhanced fluorescence luminescence instrument produced by Xingtong Medical Technology Company. Interleukin 10 (IL-10) was detected by ELISA kit from Jianglai Biological. All tests were completed within the time specified in the kit instructions, and the relevant operating procedures of the experiment were strictly followed.

### Cell Culture

Human myeloid leukemia mononuclear cells (THP-1, Huatuo Biotech, China) were cultured in RPMI 1640 medium (Thermo Fisher, USA), supplemented with 10% fetal bovine serum (FBS, Biolnd, Israel). THP-1 cells were stimulated with PMA (100 ng/ml, Sigma-Aldrich, USA) for 24 h to differentiate into M0 type macrophages. Mouse murine macrophage cells (RAW264.7, Huatuo Biotech, China) were cultured in DMEM (Thermo Fisher, USA) supplemented with 10% FBS (Sijiqing, China). The cells above were cultured at 37°C in a humidified incubator with an atmosphere containing 5% CO_2_.

### Flow Cytometry

Flow cytometry was used to detect the levels of cytokines in the supernatant. THP-1 and RAW264.7 cells were pretreated with GDF15 recombinant protein (rGDF15) (50, 100, 150, and 200 ng/ml) (R&D Systems, USA) for 0.5 h, then stimulated with lipopolysaccharide (LPS) (2μg/ml, Sigma-Aldrich, USA) for 8 h. The supernatant of culture medium was collected, 50 µl of culture medium supernatant was absorbed and mixed with capture microspheres in the ratio of 1:1, and then 50 µl of PE labeled capture antibody was added to the mixed solution. After that, the final solution was incubated in the dark at room temperature for 2.5 h. Then the IL-6, tumor necrosis factor-α (TNF-α), monocyte chemoattractant protein 1 (MCP-1), and IL-10 in the supernatant were detected by FACS Calibur. The detection kit was purchased from BD company, USA. Three independent repeated experiments were carried out.

### RNA Extraction and qPCR

The mRNA level of macrophage polarization markers was detected by qPCR. The cells were seeded in six-well plates and pretreated with rGDF15 for 0.5 h, then stimulated with LPS for 8 h. Total RNA was extracted with Trizol (Sigma-Aldrich, USA), cDNA was synthesized by reverse transcription reagent (TaKaRa, Japan), and the relative expression of CD80 and CD163 was detected by qPCR. The internal reference group was GAPDH, and the results were expressed as 2^−ΔΔCt^. The above experiments were repeated three times independently. The sequence of primers was shown in **Supplementary Table S1**.

### Detection of Phagocytosis and Bactericidal Function

The cells were inoculated into two 12-well plates and pretreated with PBS and rGDF15. After 24 h, the cells were infected with *Escherichia coli* (*E. coli*) for 1 h (the ratio of bacteria to cells was 20). Then, the cells were washed three times with PBS, and the extracellular *E. coli* was removed by adding gentamicin (10 μg/ml). After 0.5 h, a 12-well plate was taken out and washed with PBS for 3 times, then the cells were broken by pure water, and 100 µl cell crushing solution diluted for 10 times with PBS was coated on LB agar plates. Place the LB agar plates in 37°C incubator overnight, and count the colonies, which is the number of bacteria swallowed by cells; after 6 h, take out another 12 well plate and carry out the same operation as above, which is the number of bacteria remaining after cells phagocytize bacteria and play the bactericidal function. Sterilization ratio = (the colony count of 0.5 h – the colony count of 6 h)/the colony count of 0.5 h. The above experiments were repeated three times independently.

### Western Blot

THP-1 and RAW264.7 cells were seeded into a 6 cm culture dish and stimulated with LPS (0.5, 1.0, 2.0, and 4.0 μg/ml) for 8 h. The cells were lysed with RIPA lysate and protease inhibitor PMSF (biyuntian company, China) in a mixture of 100:1 for 20 min, then use an ultrasonic cell disruptor to further disrupt the cells, centrifuge at 4°C, 20000 rpm/min for 20 min, and add the supernatant to the SDS loading buffer, cooking the sample at 100°C for 10 min. Then, 30 μg of the whole-cell lysates were fractionated by 10% SDS-PAGE and transferred onto a nitrocellulose membrane. For Western blotting, membranes were blocked with 5% nonfat milk dissolved in TBST for an hour and then incubated with the primary antibodies specific for GDF15 (Bioss, China), the blots were incubated with HRP-conjugated secondary antibodies (Servicebio, China) for 1 h at room temperature, and the proteins were visualized by enhanced chemiluminescence (ECL) detection reagents (Thermo Fisher, USA) according to the manufacturer’s instruction, and the above concentration dependent experiments were repeated three times independently. At the same time, we continue to inoculate THP-1 and RAW264.7 cells into 6cm culture dishes, use LPS (2 µg/ml) to stimulate the gradient time (0, 2, 4, 6, and 8 h), the method above was used to perform Western blot to detect the expression changes of GDF15 protein, too. We also pretreated THP-1 and RAW264.7 cells with rGDF15 for 0.5 h, then stimulated with LPS, and continued to culture for 0.5 h to detect the expression of JAK1, phospho-JAK1, STAT3, phospho-STAT3, NF-κB p65, and phospho-NF-κB p65 proteins (CST, USA). Finally, we also spit out the nuclear protein with the kit (Biyuntian, China) and detect the level of phospho-NF-κB p65 in the nucleus. Each of the above experiments was carried out three times independently and repeated.

### Immunofluorescence

Immunofluorescence was used to detect the nuclear translocation level of NF-κB p65. We inoculated the cells into a 6-well plate with 25 mm × 25 mm cell slides and pretreated THP-1 and RAW264.7 cells with rGDF15 for 0.5 h, then stimulated with LPS. After 0.5 h of continuous culture, cells were fixed with 4% paraformaldehyde for 15 min, then permeabilized with Triton X-100 (Sigma-Aldrich, USA) at room temperature for 20 min, blocked with 3% BSA for 1 h afterwards, and incubated in primary anti-NF-κB p65 antibody (CST, USA) overnight at 4°C, and the secondary antibody was incubated in the next day, DAPI was used for nuclear staining and mounting. This experiment was repeated three times independently.

### ELISA

The medium supernatant of the LPS-stimulated cells using the concentration gradient and the time gradient above was collected, and the concentration of GDF15 in the medium was detected by ELISA. THP-1 cell culture medium GDF15 detection kit was purchased from R&D Company in the United States; RAW264.7 cell culture medium GDF15 detection kit was purchased from Elabscience Biotechnology Co., Ltd in the China. Operate the experiment strictly in accordance with the kit instructions. The experiment was carried out three independent repetitions.

### Statistical Analysis

Statistical analysis was performed with Excel 2019, the SPSS software 24.0, and GraphPad Prism 6.0. The measurement data uses the single-sample Kolmogorov-Smirnov method to test whether the data of each group conforms to normal distribution, the normal distribution data is expressed by the mean ± standard deviation (x– ± s), the comparison between multiple groups is performed by analysis of variance (ANOVA), and the further pairwise comparison is performed by LSD-*t* test. Pearson correlation coefficient is used to express the correlation between two groups of data; non-normal distribution data is expressed by *M (Q1, Q3)*, Kruskal-Wallis *H* test is used for comparison between multiple groups, and Mann-Whitney *U* test is used for pairwise comparison, using Spearman correlation coefficient to express the correlation between two sets of data. The enumeration data used *χ2* test. Take the receiver operating characteristic (ROC) curve for measurement data. For the ROC curve of multiple indicators, binary logistic regression analysis in SPSS 24.0 software was used to get the regression model P of multiple indicators. Then calculate the ROC curve area under the curve (AUC), AUC between 0.5 and 0.7 indicates poor diagnostic value, while 0.7–0.9 for moderate diagnostic value and over 0.9 for high diagnostic value. *P* < 0.05 indicates that the difference is statistically significant.

## Results

### Characteristics of Study Population

**Figure 1:**
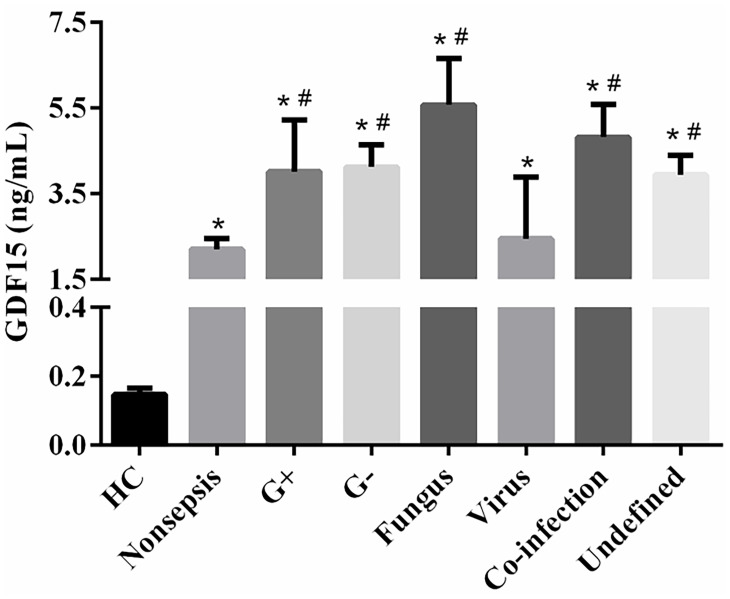
Comparison of GDF15 levels in sepsis patients infected with different pathogens. *P < 0.05, compare with control group; ^#^ *P* < 0.05, compare with nonsepsis group.

The clinical data of all subjects are shown in **Table 1**. There was no significant difference in gender and age distribution among the three groups (*P* > 0.05). The levels of inflammatory marks (WBC, CRP, SAA, IL-6, IL-10, and PCT) and GDF15 in sepsis group and nonsepsis group were higher than those in control group (*P* < 0.05), and all the above indexes in sepsis group were also higher than those in nonsepsis group (*P* < 0.05). At the same time, **Table 1** shows that the levels of RBC, Hb, and PLT in sepsis group and nonsepsis group were lower than those in control group (*P* < 0.05), and the levels of PLT in sepsis group was also lower than that in nonsepsis group. We also counted the primary infection sites of patients with sepsis. Respiratory tract, digestive tract, and urinary tract infections were the most common, with 41, 39, and 36 cases, respectively, and 16 cases were other site infections (myocardial, blood flow, etc.). Finally, we also analyzed the levels of GDF15 in patients with different pathogens (Gram-positive bacteria, Gram-negative bacteria, fungus, virus, co-infection and undefined). The results are as shown in **Figure 1**. There was no significant difference in the levels of GDF15 among patients with different pathogens, but they were higher than those in the control group. Except for viral infection, GDF15 levels in patients with other pathogens also were higher than those in nonsepsis group.

**Table 1:** Characteristics of the study population.

	Sepsis group (n = 132)	Nonsepsis group (n = 93)	Control group (n = 95)
Gender (male/female)	1.44:1 (78/54)	1.38:1 (54/39)	1.50:1 (57/38)
Age (years)	56.88 ± 14.21	56.10 ± 14.96	56.00 ± 13.94
WBC (×10^9^/L)	12.64 (7.91, 19.70)*^#^	7.14 (5.77, 10.48)*	6.24 (5.36, 7.08)
RBC (×10^12^/L)	3.56 (3.16, 4.04)*	3.80 (2.91, 4.47)*	4.54 (4.11, 4.84)
Hb (g/L)	109.50 (91.75, 122.75)*	119.00 (95.00, 136.00)*	138.00 (125.00, 153.00)
PLT (×10^9^/L)	120.00 (69.50, 207.50*^#^	187.00 (139.00, 233.00)*	221.00 (195.00, 264.00)
CRP (mg/L)	131.01 (46.08, 194.95)*^#^	10.90 (1.74, 46.72)*	0.50 (0.50, 2.73)
SAA (mg/L)	200.00 (96.60, 300.00)*^#^	22.10 (9.63, 56.75)*	5.00 (5.00, 5.00)
IL-6 (pg/mL)	62.07 (21.54, 240.55)*^#^	31.72 (12.17, 88.98)*	8.41 (6.55, 9.98)
IL-10 (pg/mL)	13.45 (7.25, 35.49)*^#^	9.34 (6.21, 17.18)*	4.67 (3.97, 5.15)
PCT (ng/mL)	18.60 (3.45, 78.80)*^#^	0.17 (0.07, 0.54)*	0.04 (0.03, 0.07)
GDF15 (ng/mL)	3.33 (1.65, 6.38)*^#^	1.06 (0.57, 3.46)*	0.10 (0.06, 0.14)
Infection site			
Respiratory tract (cases)	41	——	——
Digestive tract (cases)	39	——	——
Urinary tract (cases)	36	——	——
Other (cases)	16	——	——

*P < 0.05, compare with control group; ^#^P < 0.05, compare with nonsepsis group.

### Correlation Analysis Between GDF15 and PCT

**Figure 2:**
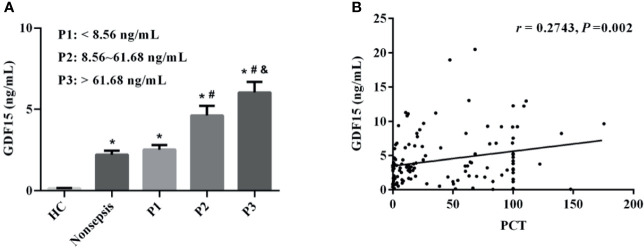
Correlation between GDF15 and PCT levels in patients with sepsis. **(A)** comparison of GDF15 levels in sepsis patients with different PCT levels; **(B)** Spearman correlation analysis of PCT and GDF15 levels. *P < 0.05, compare with HC group; ^#^ *P* < 0.05, compare with P1 group; ^&^ *P* < 0.05, compare with P2 group.

We divided sepsis patients into three groups according to the levels of PCT (the cut-off value was the interquartile distance: P1: < 8.56 ng/mL; P2: 8.56–61.68 ng/mL; P3: > 61.68 ng/mL). The concentrations of GDF15 in P1, P2, and P3 were 1.94 (1.00, 3.68) ng/mL, 3.35 (1.78, 6.52) ng/mL, and 4.94 (2.93, 9.22) ng/mL, respectively. The concentration of GDF15 in P3 is higher than that in P2 and P1, and the concentration of GDF15 in P2 is also higher than that in P1, as shown in **Figure 2A**. The Spearman correlation analysis showed that GDF15 was positively correlated with PCT, and the correlation coefficient was 0.2743 (*P* = 0.002, **Figure 2B**).

### The Relationship Between GDF15 and Sepsis Severity

**Figure 3:**
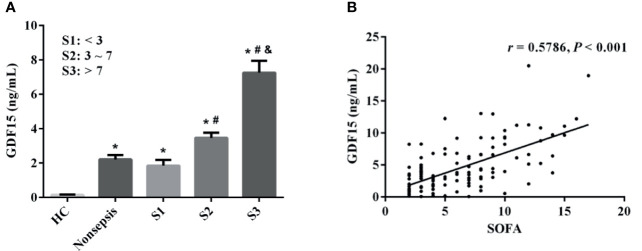
Correlation between GDF15 and SOFA scores in patients with sepsis. **(A)** comparison of GDF15 levels in sepsis patients with different SOFA scores; **(B)** Spearman correlation analysis of SOFA scores and GDF15. *P < 0.05, compare with HC group; ^#^ *P* < 0.05, compare with S1 group; ^&^ *P* < 0.05, compare with S2 group.

The sepsis patients were further divided into three groups according to SOFA scores (the cut-off value was the interquartile distance, S1: < 3 scores, S2: 3–7 scores, S3: > 7 scores). The concentrations of GDF15 in S1, S2, and S3 were 1.29 (0.85, 2.23) ng/mL, 3.19 (1.86, 4.30) ng/mL, and 6.62 (3.81, 9.88) ng/mL, respectively. As shown in **Figure 3A**, GDF15 concentration in S3 is higher than that in S2 and S1, and GDF15 concentration in S2 is also higher than that in S1. The results of Spearman correlation analysis showed that there was also a significant correlation between the two, and the correlation coefficient was 0.5786 (*P* < 0.001, **Figure 3B**).

### Correlation Analysis Between GDF15 and Laboratory Indexes

We used Spearman correlation coefficient to express the relationship between GDF15 and organ injury markers, coagulation indicators, and inflammatory indicators. The results are shown in **Table 2**. GDF15 was correlated with liver injury markers (ALB: *r* = - 0.184, *P* = 0.034; AST: *r* = 0.313, *P* < 0.001; ALT: *r* = 0.222, *P* = 0.011), as well as renal injury (Urea: *r* = 0.493, *P* < 0.001; Crea: *r* = 0.396, *P* < 0.001; UA: *r* = 0.296, *P* = 0.001) and myocardial injury markers (NT-proBNP: *r* = 0.204, *P* = 0.019; cTnI: *r* = 0.255, *P* = 0.003; CK-MB: *r* = 0.328, *P* < 0.001). Meanwhile, GDF15 was positively correlated with coagulation parameters (PLT: *r* = -0.238, *P* = 0.006; APTT: *r* = 0.203, *P* = 0.020; PT: *r* = 0.255, *P* = 0.003; TT: *r* = 0.172, *P* = 0.048). In the correlation analysis of GDF15 and inflammatory indicators, we found that GDF15 had no correlation with WBC, CRP, and SAA, but was positively correlated with IL-6 and IL-10; the correlation coefficients were 0.468 and 0.273, respectively. There was a correlation between GDF15 and multiple organ injury markers as well as inflammatory indicators.

**Table 2:** The correlation between GDF15 and organ damage markers and inflammatory indexes.

	*r*	*P*
Alb	-0.184	0.034
AST	0.313	<0.001
ALT	0.222	0.011
GGT	0.083	0.346
TBIL	0.080	0.362
DBIL	0.119	0.174
Urea	0.493	<0.001
Crea	0.396	<0.001
UA	0.296	0.001
NT-proBNP	0.204	0.019
cTnI	0.255	0.003
CK-MB	0.328	<0.001
PLT	-0.238	0.006
APTT	0.203	0.020
PT	0.255	0.003
TT	0.172	0.048
FIB	-0.088	0.317
CRP	0.065	0.461
SAA	0.007	0.933
WBC	0.126	0.149
IL6	0.468	<0.001
IL-10	0.273	0.002

### The Diagnostic Value of GDF15 for Sepsis

**Figure 4:**
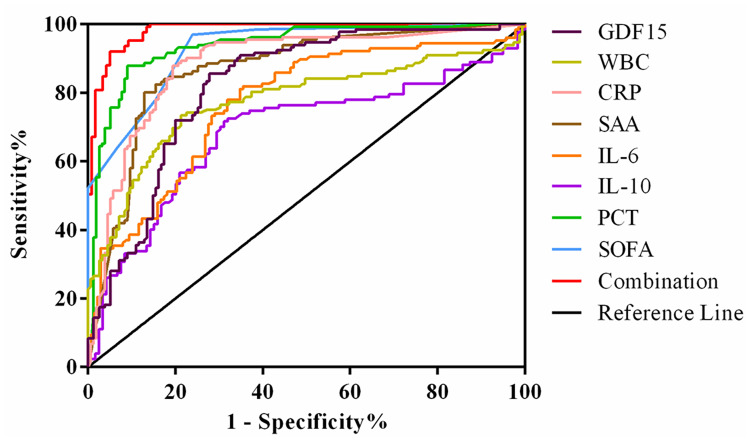
ROC curve of GDF15 combined with inflammatory markers and SOFA in diagnosis of sepsis.

We draw the ROC curve to analyze the diagnostic value of GDF15 for sepsis, and then combined it with inflammation indicators and SOFA scores to analyze the enhancement effect of GDF15 on the diagnostic value of sepsis. The results are shown in **Figure 4**. The AUC comparison of the ROC curve of each indicator was as follows: Combination (0.982) > PCT (0.937) > SOFA (0.928) > CRP (0.882) > SAA (0.867) > GDF15 (0.821) > WBC (0.775) > IL-6 (0.771) > IL-10 (0.686). Sensitivity comparison: SOFA (96.97%) > Combination (92.00%) > PCT (87.88%) = CRP (87.88%) > GDF15 (85.61%) > IL-6 (82.03%) > SAA (80.15%) > WBC (73.48%) > IL-10 (71.65%). Specificity comparison: Combination (94.96%) > PCT (90.97%) > SAA (87.10%) > CRP (80.65%) > WBC (78.71%) > SOFA (76.13%) > GDF15 (72.26%) > IL-10 (68.91%) > IL-6 (64.49%). The cut-off values of GDF15, WBC, CRP, SAA, IL-6, IL-10, PCT and SOFA were 1.18 ng/mL, 8.53×10^9^/L, 22.30 mg/L, 61.54 mg/L, 17.30 pg/mL, 6.65 pg/mL, 0.96 ng/mL and 1, respectively (**Table 3**). The above data showed that the combination of indicators has the highest diagnostic value for sepsis.

**Table 3:** ROC curve analysis of GDF15 combined with inflammatory markers and SOFA in the diagnosis of sepsis.

	AUC	Sensitivity%	Specificity%	95%CI	Youden index	Cut-off
GDF15	0.821	85.61	72.26	0.772–0.864	0.5786	1.18 ng/mL
WBC	0.775	73.48	78.71	0.722–0.822	0.5219	8.53×10^9^/L
CRP	0.882	87.88	80.65	0.839–0.917	0.6852	22.30 mg/L
SAA	0.867	80.15	87.1	0.822–0.904	0.6725	61.54 mg/L
IL-6	0.771	82.03	64.49	0.716–0.821	0.4652	17.30 pg/mL
IL-10	0.686	71.65	68.91	0.624–0.744	0.4056	6.65 pg/mL
PCT	0.937	87.88	90.97	0.902–0.962	0.7885	0.96 pg/mL
SOFA	0.928	96.97	76.13	0.892–0.955	0.731	1
Combination	0.982	92	94.96	0.957–0.995	0.8696	–

### Dynamic Monitoring of GDF15 Levels in Patients with Sepsis and Septic Shock

**Figure 5:**
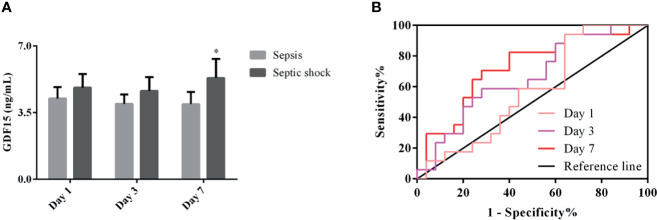
Dynamic analysis of GDF15 in sepsis group and septic shock group. **(A)** the dynamic changes of GDF15 levels in sepsis group and septic shock group; **(B)** ROC curve of dynamic changes of GDF15 in differentiating sepsis from septic shock. *P < 0.05, compare with Day 7 of sepsis group.

We divided the patients under dynamic monitoring into sepsis group and septic shock group, and analyzed the GDF15 levels of the two groups of patients on the 1st, 3rd, and 7th days after admission and treatment. The results are shown in **Figure 5A**. The GDF15 levels of the two groups of patients after admission to the hospital did not fluctuate greatly (*P* > 0.05), and the GDF15 levels of the sepsis group and the septic shock group were not significantly different on the 1st day and the 3rd day (*P* > 0.05); nevertheless, the levels of GDF15 in the septic shock group were higher than that in the sepsis group on the 7th day (*P* < 0.05). On the whole, the level of GDF15 in septic shock group showed an upward trend, while the level of GDF15 in sepsis group changed little.

### The Clinical Value of GDF15 in Distinguishing Sepsis from Septic Shock

We drew the ROC curve to further analyze the dynamic GDF15 levels to distinguish between sepsis and septic shock (**Figure 5B**). Comparison of AUC at three time points of dynamic monitoring: Day 7 (0.715) > Day 3 (0.661) > Day 1 (0.569). Sensitivity comparison: Day 1 (94.12%) > Day 7 (70.59%) > Day 3 (58.82%). Specificity comparison: Day 7 (72.00%) = Day 3 (72.00%) > Day 1 (36.00%). The cut-off values for Day 1, Day 3, and Day 7 were 2.79, 4.01, and 3.91 ng/mL, respectively, as shown in **Table 4**. Day 7 was of the highest comprehensive clinical value in differentiating sepsis and septic shock, while Day 1 has the highest sensitivity.

**Table 4:** ROC curve analysis of dynamic monitoring GDF15 in sepsis and septic shock.

	AUC	Sensitivity%	Specificity%	95%CI	Youden index	Cut-off
Day 1	0.569	94.12	36.00	0.408–0.721	0.3012	2.79 ng/mL
Day 3	0.661	58.82	72	0.499–0.800	0.3082	4.01 ng/mL
Day 7	0.725	70.59	72	0.565–0.851	0.4259	3.91 ng/mL

### Dynamic Monitoring of GDF15 Levels in Death Group and Survival Group

**Figure 6:**
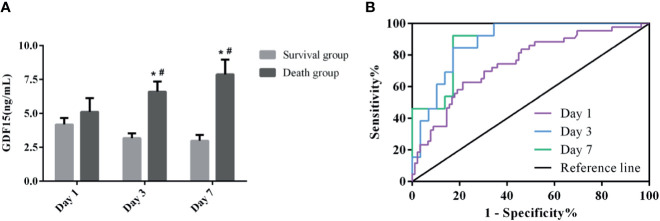
Dynamic analysis of GDF15 in survival group and death group. **(A)**: the dynamic changes of GDF15 levels in survival group and death group; **(B)**: ROC curve of dynamic changes of GDF15 on prognosis of sepsis. *P < 0.05, compare with the same day of survival group; ^#^ *P* < 0.05, compare with Day 1 of the same group.

At the same time, we also divided the patients under dynamic monitoring into survival group and death group, and analyzed the GDF15 levels of the two groups on the 1st, 3rd, and 7th day after admission (**Figure 6A**). The GDF15 levels in the survival group decreased after admission, but the difference was not significant (*P* > 0.05). Interestingly, in the death group, the GDF15 levels increased gradually instead on the 1st, 3rd and 7th day, which were 3.77 (2.77, 7.45) ng/mL, 6.63 (4.00, 8.39) ng/mL, and 6.77 (4.34, 11.69) ng/mL, respectively. Compared with the 1st day, the GDF15 levels on the 3rd and 7th day was increased (*P* < 0.05). At the same time, the GDF15 levels of death group were higher than that of survival group on the 3rd and 7th day (*P* < 0.05), but there was no significant difference on the 1st day. We can clearly observe that the GDF15 of the survival group showed a decreasing trend, while the GDF15 of the death group showed an increasing trend.

### Prognostic Value of GDF15 in Patients With Sepsis

**Figure 6B** shows the ROC curve of the clinical value of dynamic GDF15 levels on the prognosis of sepsis. Comparison of AUC at three time points of dynamic monitoring: Day 7 (0.897) > Day 3 (0.886) > Day 1 (0.748). Sensitivity comparison: Day 7 (92.31%) > Day 3 (84.62%) > Day 1 (62.79%). Specificity comparison: Day 7 (82.76%) = Day 3 (82.76%) > Day 1 (78.65%). The cut-off values for Day 1, Day 3 and Day 7 were 4.38, 3.96, and 3.76 ng/mL, respectively, as shown in **Table 5**. The prognostic value of GDF15 level of Day 7 in sepsis was better than that of the other two points of time.

**Table 5:** ROC curve analysis of dynamic monitoring GDF15 in the prognosis of sepsis.

	AUC	Sensitivity%	Specificity%	95%CI	Youden index	Cut-off
Day 1	0.748	62.79	78.65	0.665–0.820	0.4144	4.38 ng/mL
Day 3	0.886	84.62	82.76	0.750–0.963	0.6737	3.96 ng/mL
Day 7	0.897	92.31	82.76	0.763–0.969	0.7507	3.76 ng/mL

### The Expression of GDF15 in THP-1 and RAW264.7 Cells Stimulated by LPS

**Figure 7:**
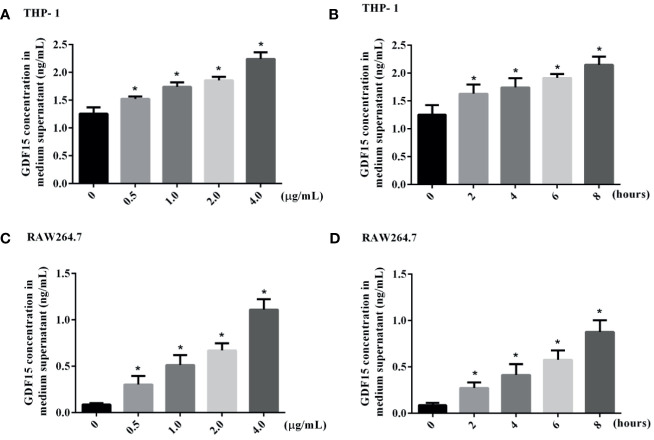
The concentration of GDF15 in the culture medium of stimulated by LPS was tested by ELISA. **(A)** THP-1 cells stimulated by gradient concentration of LPS; **(B)** THP-1 cells stimulated by gradient time of LPS; **(C)** RAW264.7 cells stimulated by gradient concentration of LPS; **(D)** RAW264.7 cells stimulated by gradient time of LPS. *P < 0.05, compare with the blank group.

**Figure 8:**
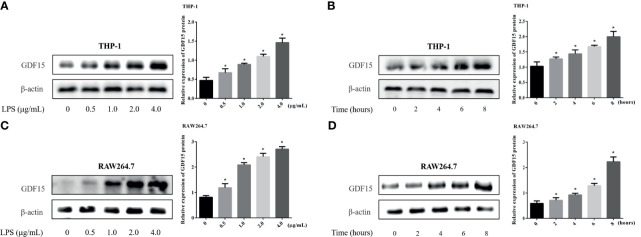
Changes of GDF15 protein expression in THP-1 and RAW264.7 cells stimulated by LPS. The protein expression was detected by Western blot, and the picture showed the protein bands and their quantitative analysis results. **(A)** THP-1 cells stimulated by gradient concentration of LPS; **(B)** THP-1 cells stimulated by gradient time of LPS; **(C)** RAW264.7 cells stimulated by gradient concentration of LPS; **(D)** RAW264.7 cells stimulated by gradient time of LPS. *P < 0.05, compare with the blank group.

We used LPS to stimulate THP-1 and RAW264.7 cells to imitate an inflammation model *in vitro*, and analyzed both of the concentration of GDF15 in medium supernatant and the GDF15 expression of cells in the inflammatory state. The results are shown in **Figures 7** and **8**. Compared with the blank group, the concentration of GDF15 in medium supernatant and the GDF15 expression of cells increased after LPS stimulation, and with the increase of the LPS concentration and the prolongation of the stimulation time, the concentration of GDF15 as well as the GDF15 expression of cells showed an upward trend.

### GDF15 Alleviate the Inflammatory Response of THP-1 and RAW264.7 Cells Stimulated by LPS

**Figure 9:**
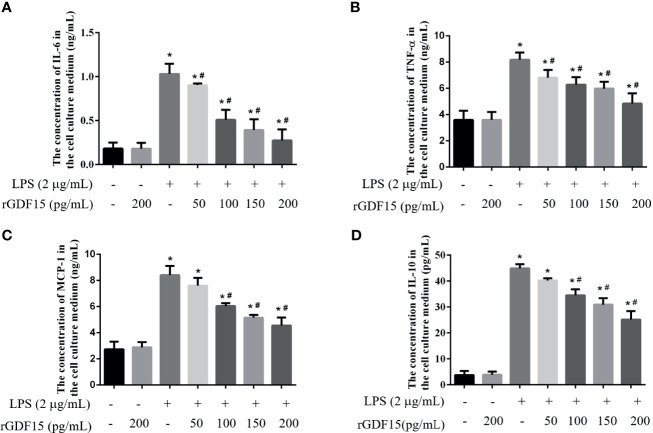
The levels of IL-6 **(A)**, TNF-α **(B)**, MCP-1 **(C)**, and IL-10 **(D)** were changed after pretreatment with rGDF15 in inflammatory state of THP-1 cells. These cytokines were detected by flow cytometry. *P < 0.05, compare with the blank group; ^#^ *P* < 0.05, compare with the LPS group.

**Figure 10:**
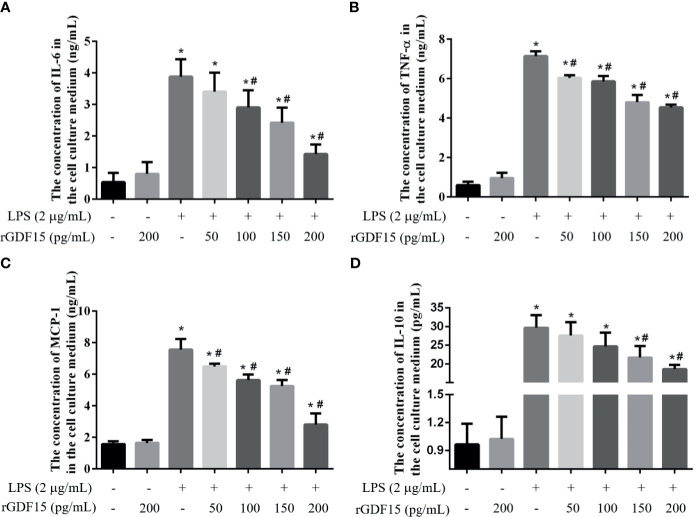
The levels of IL-6 **(A)**, TNF-α **(B)**, MCP-1 **(C)**, and IL-10 **(D)** were changed after pretreatment with rGDF15 in inflammatory state of RAW264.7 cells. These cytokines were measured by flow cytometry. *P < 0.05, compare with the blank group; ^#^ *P* < 0.05, compare with the LPS group.

Inflammation is a sign of sepsis, and the levels of inflammatory factors can reflect the degree of inflammation. We pretreated different concentrations of rGDF15 to the cells stimulated by LPS, and analyzed the changes in the levels of inflammatory factors in the medium supernatant. Compared with the blank group, the levels of inflammatory factors like IL-6, TNF-α, MCP-1, and IL-10 increased significantly after the addition of LPS, while pretreatment with rGDF15, the concentrations of the mentioned cytokines above decreased on the contrary (**Figures 9**, **10**). The higher the concentration of pretreated rGDF15, the lower the decline of inflammatory factors. This phenomenon shows that GDF15 can reduce the increase of cytokines in inflammatory state.

### Effect of GDF15 on Polarization of THP-1 and RAW264.7 Cells

**Figure 11:**
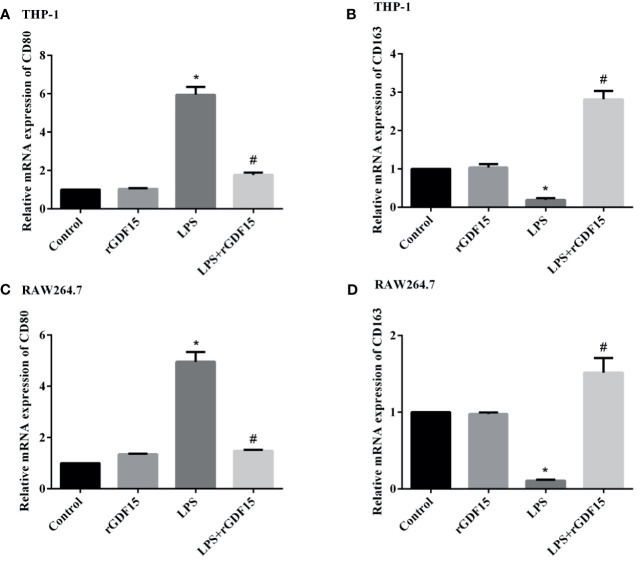
Effect of rGDF15 on macrophage polarization. The expression levels of polarization markers mRNA were detected by qPCR. The relative mRNA expression of CD80 **(A)** and CD163 **(B)** in THP-1 cells; the relative mRNA expression of CD80 **(C)** and CD163 **(D)** in RAW264.7 cells. *P < 0.05, compare with the control group; ^#^ *P* < 0.05, compare with the LPS group.

We detected the mRNA expression of M1 and M2 polarization markers respectively, and the results are shown in **Figure 11**. We found that the mRNA expression of CD80 in cells stimulated by LPS was significantly increased, the pretreatment of rGDF15 can downregulate the increase of CD80; interestingly, the mRNA expression of CD163 in cells stimulated by LPS decreased, and it could be upregulated by pretreatment with rGDF15. The above results indicate that pretreatment of rGDF15 can inhibit the M1 type polarization of macrophages and promote the M2 type polarization on the contrary.

### GDF15 Improves the Phagocytosis and Bactericidal Function of Macrophages

**Figure 12:**
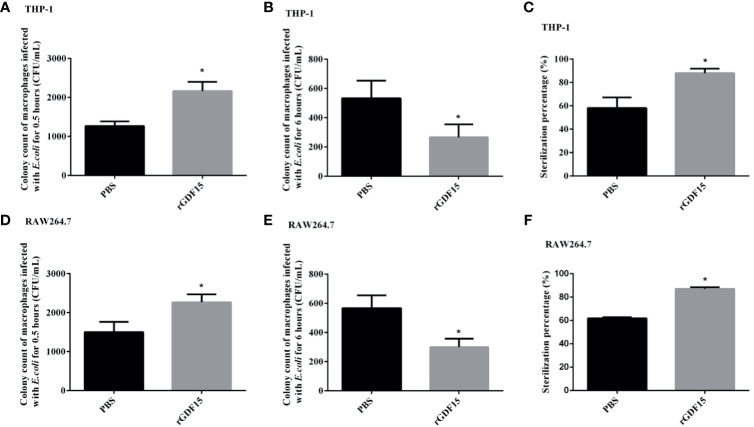
The phagocytosis of macrophages was identified by *E. coli* infection, and the bactericidal function was determined by gentamicin protection assay. RGDF15 enhanced the phagocytosis of THP-1 **(A)** and RAW264.7 **(D)** cells; it also improved the bactericidal function of THP-1 **(B, C)** and RAW264.7 **(E, F)** cells. *P < 0.05, compare with the PBS group.

We further explored the effect of GDF15 on the phagocytosis and bactericidal function of macrophages. The results showed that at 0.5 h of *E. coli* infection, the colony count of macrophages in the GDF15 treatment group was significantly higher than that of the PBS treatment group (*P* < 0.05), that is, the phagocytic function of macrophages in the GDF15 treatment group was higher than that of the PBS treatment group; at the same time, we also observed the sterilization ratio of the GDF15 treatment group was much higher than that of the PBS treatment group, indicating that the macrophages of the GDF15 treatment group had stronger bactericidal function (**Figure 12**).

### GDF15 Reduces the Phosphorylation Levels of JAK1/STAT3 Pathway Protein

**Figure 13:**
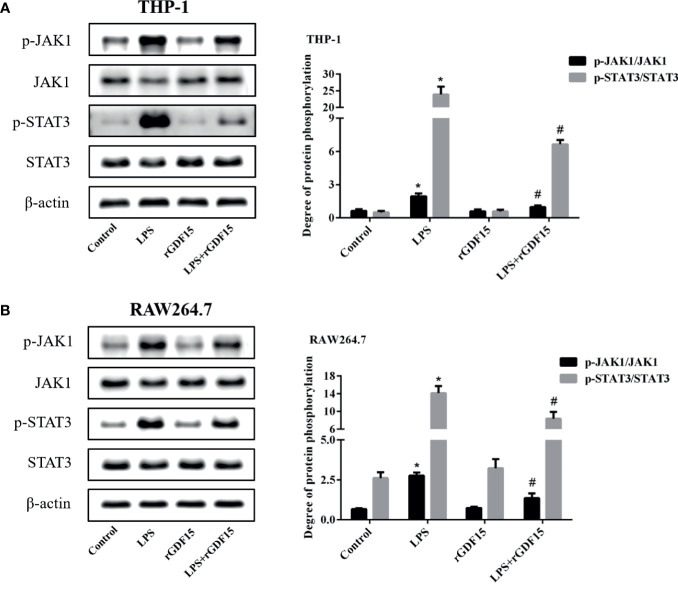
Phosphorylation of JAK1/STAT3 pathway in THP-1 **(A)** and RAW264.7 **(B)** cells pretreated with rGDF15 in inflammatory state was detected by Western blot; the protein band diagram and quantitative analysis results were shown here. *P < 0.05, compare with the control group; ^#^ *P* < 0.05, compare with the LPS group.

After LPS stimulated THP-1 and RAW264.7 cells, the phosphorylation levels of JAK1/STAT3 pathway proteins were significantly increased, while the phosphorylation levels of JAK1/STAT3 pathway proteins were decreased after pretreatment with rGDF15 (**Figure 13**). Obviously, the pretreatment of rGDF15 reduced the phosphorylation of JAK1/STAT3.

### GDF15 Reduces the Level of Nuclear Translocation of NF-κB p65

**Figure 14:**
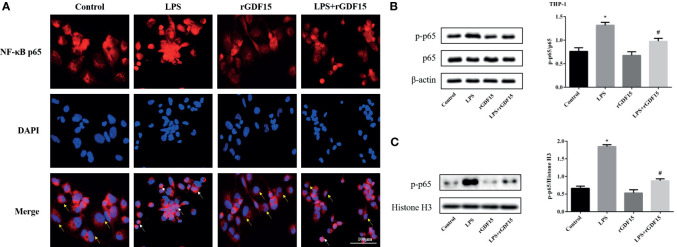
Nuclear translocation of NF-κB p65 in THP-1 cells pretreated with rGDF15 in inflammatory state was detected by immunofluorescence **(A)**. Scale bar = 100 μm. Red fluorescence marked NF-κB p65 and blue fluorescence marked nucleus. Yellow arrows indicated nuclear translocation negative cells; white arrows indicated nuclear translocation positive cells. The NF-κB p65 phosphorylation level of THP-1 cells pretreated with rGDF15 in inflammatory state was detected by Western blot **(B)**. The expression of p-p65 in the nucleus was detected by Western blot **(C)**. *P < 0.05, compare with the control group; ^#^ *P* < 0.05, compare with the LPS group.

**Figure 15:**
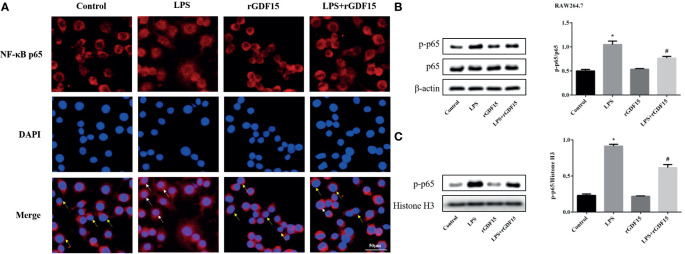
Nuclear translocation of NF-κB p65 in RAW264.7 cells pretreated with rGDF15 in inflammatory state was detected by immunofluorescence **(A)**. Scale bar = 50 μm. Red fluorescence marked NF-κB p65 and blue fluorescence marked nucleus. Yellow arrows indicated nuclear translocation negative cells; white arrows indicated nuclear translocation positive cells. The NF-κB p65 phosphorylation level of RAW264.7 cells pretreated with rGDF15 in inflammatory state was detected by Western blot **(B)**. The expression of p-p65 in the nucleus was detected by Western blot **(C)**. *P < 0.05, compare with the control group; ^#^ *P* < 0.05, compare with the LPS group.

As shown in **Figures 14**, **15**, compared with the control group, we observed that there was an obvious nuclear translocation of NF-κB p65 in LPS group. While THP-1 and RAW264.7 cells were pretreated with rGDF15 for 0.5 h and then stimulated with LPS, the phenomenon of nuclear translocation was suppressed. At the same time, we used Western blot to quantitatively analyze the phosphorylation of NF-κB p65 in cells and nuclei, and the results showed that the phosphorylation of NF-κB p65 was inhibited by rGDF15 pretreatment in inflammatory state.

## Discussion

Sepsis is caused by various causes of immune dysfunction and excessive release of inflammatory mediators, leading to extensive tissue damage and multiple organ dysfunction, which has a poor prognosis (28). In recent years, the incidence rate and mortality rate of sepsis has decreased slightly with the improvement of medical conditions, but it is still the main cause of global health damage (2). In order to improve the prognosis of sepsis, early identification and intervention is particularly important, so it is urgent to find more biomarkers and explore the regulatory mechanism of inflammatory response in sepsis. Our study shows that GDF15 is increased in patients with sepsis, and it is correlated with PCT, SOFA scores, and many laboratory indexes. ROC curve also indicates that GDF15 has good diagnostic value for sepsis; meanwhile, the dynamic monitoring of GDF15 levels is of high prognostic value for patients with sepsis, and we speculate that GDF15 may be a biomarker of sepsis. Finally, we further established macrophage inflammation model and found that rGDF15 could reduce the levels of cytokines secreted by macrophages in inflammatory state and it could also inhibit the M1 type polarization as well as the phosphorylation levels of JAK1/STAT3 signaling pathway and nuclear translocation of NF-κB p65. What is more, rGDF15 could improve phagocytosis and bactericidal function of macrophages, which supports the conclusion that GDF15 plays a protective role in sepsis together with the results above.

At present, there are more and more studies on GDF15 in various diseases. Studies suggest that the increase of serum GDF15 concentration is related to the development and progress of cardiovascular disease (29). The analysis of more than 100 original studies (30–34) shows that GDF15 has higher value in predicting all-cause mortality in patients with heart failure than traditional cardiovascular risk factors and biomarkers. Other studies have proposed that the expression of GDF15 in the whole nephron is weak in physiological state, but the concentration of plasma GDF15 rises sharply before acute kidney injury (35), scholars believe that it may be an early sign of subclinical kidney injury, but it cannot be detected using conventional clinical parameters. They also discovered that in the stage of renal endothelial dysfunction, the increase of GDF15 precedes the increase of microalbumin (36, 37). All the above studies show that GDF15 is of great significance in the progress of disease, which may be a comprehensive indicator and has considerable clinical application value (38). Similarly, there are a few studies on GDF15 in sepsis. For example, an article proposed that GDF15 could be induced by immune and nonimmune cells, which controlled hepatic sympathetic outflow and maintained systemic triglyceride level through adrenergic signal and cardiac function to improves the body’s tolerance to inflammation (17). The perspective of this study is different from the above, we analyzed the clinical value of GDF15 for sepsis and its regulation of inflammatory signal pathways. We have observed that serum GDF15 levels in patients with sepsis are elevated, which is consistent with the results of previous studies (13–17). As we all know, PCT is the most common laboratory indicator used in sepsis. Its concentration will increase significantly in bacterial infection, but not in viral infection. Our study found that GDF15 will increase in sepsis caused by various pathogens, which is one aspect of GDF15 superior to PCT. At the same time, we observed that the concentration of GDF15 in sepsis patients with different PCT levels is also different. Patients with high PCT levels are often accompanied by high GDF15 levels, and there is a weak positive correlation between the two serum biomarkers. SOFA score system is the standard for sepsis diagnosis stipulated in the latest sepsis definition (1), and is related to the condition of critically ill patients. The higher SOFA score, the more serious the condition (39). Our research results show that SOFA score is significantly positively correlated with GDF15, so we speculate that the higher GDF15 score, the more serious the condition is, this conclusion is consistent with the studies of Lee et al. (14) and Buendgens et al. (15).

In the correlation analysis of GDF15 and laboratory indicators, we observed that GDF15 was correlated with biomarkers of liver, kidney, and heart damage, which was not difficult to explain, because previous studies found that GDF15 had changes in many organ-related diseases (40–42), and the course of sepsis involved multiple organ injuries, so this result was in line with expectations, which is also the same as the previous findings (15, 43). The coagulation system also plays an important role in the pathogenesis of sepsis (44). It promotes each other with inflammatory reaction, and together constitutes a key factor in the occurrence and development of sepsis. Interestingly, GDF15 is also correlated with coagulation indicators, which further confirms that GDF15 is a comprehensive indicator (43), which can reflect the overall state of the body. There is no study on GDF15 and coagulation function up to now, which is worth further research. Some studies have pointed out that cytokines can also induce the secretion of GDF15 (45). This study found that GDF15 is positively correlated with IL-6 and IL-10, which may be due to the rapid increase of cytokines after the cytokine storm in sepsis.

Several studies have analyzed the diagnostic and prognostic value of GDF15 in sepsis (13–15), but no dynamic monitoring analysis has been carried out. Once sepsis is diagnosed, the patients are usually sent to ICU for intensive care, the changes of various indicators of the patients were needed to pay close attention to. We carried out dynamic monitoring of GDF15 of the research population and compared them in groups. The results show that the dynamic monitoring of GDF15 has little difference between the general sepsis group and the septic shock group, but the trend is consistent with the severity of the disease. The levels of GDF15 in the shock group increased even after admission, while the general sepsis group showed a downward trend, which was more obvious in the survival group and the death group. The levels of GDF15 in the survival group began to decrease after admission, while the death group began to further increase on the contrary, which was higher than the survival group, this can be used as the basis for clinicians to judge the patient’s condition. If the concentration of GDF15 remains unchanged or begins to rise after treatment, clinicians should pay more close attention to the patient’s condition and make timely intervention, which may improve the survival rate of patients. Combined with the ROC curve results, we think that the dynamic monitoring of GDF15 has good clinical application value. Unfortunately, our study is only a single-center study in a short period of time, so the cut-off value obtained by ROC curve analysis in this paper is different from that reported by other researches (13–15), and cannot be used as a good clinical reference value. Therefore, we suggest that qualified research groups can conduct larger-scale research and put GDF15 into clinical application as soon as possible.

Some scholars previously reported that the expression of intracellular GDF15 increased with the force application (46). In this study, LPS was used to stimulate macrophages, and it was also observed that the expression of intracellular GDF15 increased, while the concentration of GDF15 in the culture medium also increased, which echoed the results of our serology. At the same time, with the extension of LPS stimulation time, the level of GDF15 showed a further upward trend. Our studies also showed that pretreatment with rGDF15 could reduce the levels of cytokines stimulated by LPS, which indicated that GDF15 played a protective role. The results above were the same as the conclusion put forward by Luan et al. (17). The imbalance of inflammatory immune response in sepsis is related to the complex interaction of a variety of signaling pathways, which are abnormally activated or inhibited, leading to regulatory dysfunction (47). Studies suggest that macrophage polarization is involved in the occurrence, development and prognosis of many inflammatory diseases (48). M0 macrophages can be divided into pro-inflammatory M1 type and anti-inflammatory M2 type according to phenotypic and functional changes. M1 type macrophages highly express molecules such as IL-6, TNF-α, and CD80, while M2 type macrophages highly express CD163, CD206, and TGF-β. This study found that macrophages pretreated with rGDF15 promoted the expression of M2 type markers and inhibited the expression of M1 type markers in an inflammatory state, which further confirmed that GDF15 exerted an anti-inflammatory effect. Phagocytosis is also closely related to inflammation and immune response. To some extent, low phagocytosis means a high risk of infection and poor clinical outcomes (49). In the late stage of sepsis, the phagocytic function of macrophages and monocytes are damaged and the immune system is paralyzed, resulting in the failure of timely removal of pathogens in patients and aggravating the disease. Therefore, it is of great significance to improve the phagocytic function of phagocytes in the case of sepsis. Previous research reported that GDF15 can enhance the phagocytosis of dendritic cells in tumor environment (50). We applied gentamicin protection assay to explore the effect of GDF15 on the phagocytosis and bactericidal function of macrophages. Interestingly, the results showed that GDF15 could improve the phagocytic function of macrophages, and its bactericidal function was also improved, which undoubtedly suggested that GDF15 was promising as an important therapeutic target for sepsis, but the specific mechanism needs to be further explored.

Overexpression of inflammatory mediators in sepsis is associated with a variety of signaling pathways, such as JAK/STAT. The activation of JAK/STAT signaling pathway will lead to the accumulation of a large number of pro-inflammatory cytokines and aggravate inflammation (51). In recent years, JAK1/STAT3 signaling pathway has attracted more and more attention in the regulation of inflammatory response in sepsis (52, 53). In this study, we observed that JAK1/STAT3 signaling pathway was activated after LPS stimulation in THP-1 and RAW264.7 cells, but rGDF15 pretreatment could effectively inhibit its phosphorylation, which indicated that GDF15 could reduce the inflammatory response, which was consistent with our previous results. Many literatures have proved that the transcription activation of NF-κB gene is considered as the most important step in LPS induced signal cascade reaction. After NF-κB is activated, IκB phosphorylation degradation leads to the phosphorylation and transfer from cytoplasm to nucleus of NF-κB p65 subunit (54). The results also revealed that the pretreatment of rGDF15 could reduce the nuclear translocation and the phosphorylation of NF-κB p65 stimulated by LPS, and further confirmed that GDF15 can reduce the inflammatory response.

## Conclusion

In summary, this study found that the increase of GDF15 in serum of patients was closely related to organ injury and severity of sepsis. Dynamic monitoring of GDF15 can indicate the trend of the disease and has a good diagnosis and prognostic value, even may be a biomarker of sepsis in the future. We consider that GDF15 plays a protective role in sepsis; it can inhibit the M1 polarization of macrophages as well as the phosphorylation of JAK1/STAT3 signaling pathway and nuclear translocation of NF-κB p65 to alleviate inflammation. It can also enhance the phagocytosis and bactericidal function of macrophages and promote the clearance of pathogens.

## Data Availability Statement

The original contributions presented in the study are included in the article/**Supplementary Material**. Further inquiries can be directed to the corresponding author.

## Ethics Statement

The studies involving human participants were reviewed and approved by The Ethics Committee of the Renmin Hospital of Wuhan University. Written informed consent for participation was not required for this study in accordance with the national legislation and the institutional requirements.

## Author Contributions

HL and DT designed and performed the study and wrote the manuscript. PZ was responsible for the revision and review of the paper. JC provided the ideas of the manuscript. YH and XC were responsible for collecting data. All authors contributed to the article and approved the submitted version.

## Funding

This research was funded by National Natural Science Foundation of China (81773444) and Natural Science Foundation of Hubei Province (2019CFC846).

## Conflict of Interest

The authors declare that the research was conducted in the absence of any commercial or financial relationships that could be construed as a potential conflict of interest.

## Publisher’s Note

All claims expressed in this article are solely those of the authors and do not necessarily represent those of their affiliated organizations, or those of the publisher, the editors and the reviewers. Any product that may be evaluated in this article, or claim that may be made by its manufacturer, is not guaranteed or endorsed by the publisher.

## References

[1] Singer M Deutschman CS Seymour CW Shankar-Hari M Annane D Bauer M. The Third International Consensus Definitions for Sepsis and Septic Shock (Sepsis-3). JAMA (2016) 315:801–10. 10.1001/jama.2016.0287 PMC4968574 26903338

[2] Gańczak M Miazgowski T Kożybska M Kotwas A Korzeń M Rudnicki B. Changes in Disease Burden in Poland Between 1990-2017 in Comparison With Other Central European Countries: A Systematic Analysis for the Global Burden of Disease Study 2017. PloS One (2020) 15:e0226766. 10.1371/journal.pone.0226766 32119685 PMC7051048

[3] Paoli CJ Reynolds MA Sinha M Gitlin M Crouser E. Epidemiology and Costs of Sepsis in the United States-An Analysis Based on Timing of Diagnosis and Severity Level. Crit Care Med (2018) 46:1889–97. 10.1097/CCM.0000000000003342 PMC6250243 30048332

[4] Gai L Tong Y Yan BQ. Research on the Diagnostic Effect of PCT Level in Serum on Patients With Sepsis Due to Different Pathogenic Causes. Eur Rev Med Pharmacol Sci (2018) 22:4238–42. 10.26355/eurrev_201807_15418 30024613

[5] Zhu Y Zhu J Lu C Zhang Q Xie W Sun P. Identification of Protein Abundance Changes in Hepatocellular Carcinoma Tissues Using PCT-SWATH. Proteomics Clin Appl (2019) 13:e1700179. 10.1002/prca.201700179 30365225

[6] Kamat IS Ramachandran V Eswaran H Guffey D Musher DM. Procalcitonin to Distinguish Viral From Bacterial Pneumonia: A Systematic Review and Meta-Analysis. Clin Infect Dis (2020) 70:538–42. 10.1093/cid/ciz545 31241140

[7] Breit SN Johnen H Cook AD Tsai VW Mohammad MG Kuffner T. The TGF-β Superfamily Cytokine, MIC-1/GDF15: A Pleotrophic Cytokine With Roles in Inflammation, Cancer and Metabolism. Growth Factors (2011) 29:187–95. 10.3109/08977194.2011.607137 21831009

[8] Ho JE Lyass A Courchesne P Chen G Liu C Yin X. Protein Biomarkers of Cardiovascular Disease and Mortality in the Community. J Am Heart Assoc (2018) 7:e008108. 10.1161/JAHA.117.008108 30006491 PMC6064847

[9] George M Jena A Srivatsan V Muthukumar R Dhandapani VE. GDF 15–A Novel Biomarker in the Offing for Heart Failure. Curr Cardiol Rev (2016) 12:37–46. 10.2174/1573403x12666160111125304 26750722 PMC4807717

[10] Fuernau G Poenisch C Eitel I de Waha S Desch S Schuler G. Growth-Differentiation Factor 15 and Osteoprotegerin in Acute Myocardial Infarction Complicated by Cardiogenic Shock: A Biomarker Substudy of the IABP-SHOCK II-Trial. Eur J Heart Fail (2014) 16:880–7. 10.1002/ejhf.117 24903195

[11] Hansen ES Hindberg K Latysheva N Aukrust P Ueland T Hansen JB. Plasma Levels of Growth Differentiation Factor 15 are Associated With Future Risk of Venous Thromboembolism. Blood (2020) 136:1863–70. 10.1182/blood.2019004572 32645137

[12] Myojin Y Hikita H Sugiyama M Sasaki Y Fukumoto K Sakane S. Hepatic Stellate Cells in Hepatocellular Carcinoma Promote Tumor Growth *Via* Growth Differentiation Factor 15 Production. Gastroenterology (2021) 160:1741–54. 10.1053/j.gastro.2020.12.015 33346004

[13] Myhre PL Prebensen C Strand H Røysland R Jonassen CM Rangberg A. Growth Differentiation Factor 15 Provides Prognostic Information Superior to Established Cardiovascular and Inflammatory Biomarkers in Unselected Patients Hospitalized With COVID-19. Circulation (2020) 142:2128–37. 10.1161/CIRCULATIONAHA.120.050360 PMC7688084 33058695

[14] Lee CW Kou HW Chou HS Chou HH Huang SF Chang CH. A Combination of SOFA Score and Biomarkers Gives a Better Prediction of Septic AKI and in-Hospital Mortality in Critically Ill Surgical Patients: A Pilot Study. World J Emerg Surg (2018) 13:41–7. 10.1186/s13017-018-0202-5 PMC6131912 30214469

[15] Buendgens L Yagmur E Bruensing J Herbers U Baeck C Trautwein C. Growth Differentiation Factor-15 Is a Predictor of Mortality in Critically Ill Patients With Sepsis. Dis Markers (2017) 12:5271203. 10.1155/2017/5271203 PMC5664246 29180833

[16] Mueller T Leitner I Egger M Haltmayer M Dieplinger B. Association of the Biomarkers Soluble ST2, Galectin-3 and Growth-Differentiation Factor-15 With Heart Failure and Other Non-Cardiac Diseases. Clin Chim Acta (2015) 445:155–60. 10.1016/j.cca.2015.03.033 25850080

[17] Luan HH Wang A Hilliard BK Carvalho F Rosen CE Ahasic AM. GDF15 Is an Inflammation-Induced Central Mediator of Tissue Tolerance. Cell (2019) 178:1231–44. 10.1016/j.cell.2019.07.033 PMC6863354 31402172

[18] Fujita Y Ito M Ohsawa I. Mitochondrial Stress and GDF15 in the Pathophysiology of Sepsis. Arch Biochem Biophys (2020) 696:108668. 10.1016/j.abb.2020.108668 33188737

[19] Khamri W Abeles RD Hou TZ Anderson AE El-Masry A Triantafyllou E. Increased Expression of Cytotoxic T-Lymphocyte-Associated Protein 4 by T Cells, Induced by B7 in Sera, Reduces Adaptive Immunity in Patients With Acute Liver Failure. Gastroenterology (2017) 153:263–76. 10.1053/j.gastro.2017.03.023 PMC5516432 28363639

[20] Shen Q Zhang Q Shi Y Shi Q Jiang Y Gu Y. Tet2 Promotes Pathogen Infection-Induced Myelopoiesis Through mRNA Oxidation. Nature (2018) 554:123–7. 10.1038/nature25434 29364877

[21] Teng JF Wang K Jia ZM Guo YJ Guan YW Li ZH. Lentivirus-Mediated Silencing of Src Homology 2 Domain-Containing Protein Tyrosine Phosphatase 2 Inhibits Release of Inflammatory Cytokines and Apoptosis in Renal Tubular Epithelial Cells *Via* Inhibition of the TLR4/NF-kB Pathway in Renal Ischemia-Reperfusion Injury. Kidney Blood Press Res (2018) 43:1084–103. 10.1159/000491565 29991025

[22] Zhu Y Wan N Shan X Deng G Xu Q Ye H. Celastrol Targets Adenylyl Cyclase-Associated Protein 1 to Reduce Macrophages-Mediated Inflammation and Ameliorates High Fat Diet-Induced Metabolic Syndrome in Mice. Acta Pharm Sin B (2021) 11:1200–12. 10.1016/j.apsb.2020.12.008 PMC8148064 34094828

[23] Vanderhaeghen T Wallaeys C Libert C. Turning a Pathogen Protein Into a Therapeutic Tool for Sepsis. EMBO Mol Med (2021) 13:e13589. 10.15252/emmm.202013589 33332738 PMC7799353

[24] Li M Song K Huang X Fu S Zeng Q. GDF−15 Prevents LPS and D−Galactosamine−Induced Inflammation and Acute Liver Injury in Mice. Int J Mol Med (2018) 42:1756–64. 10.3892/ijmm.2018.3747 29956733

[25] Abulizi P Loganathan N Zhao D Mele T Zhang Y Zwiep T. Growth Differentiation Factor-15 Deficiency Augments Inflammatory Response and Exacerbates Septic Heart and Renal Injury Induced by Lipopolysaccharide. Sci Rep (2017) 7:1037–43. 10.1038/s41598-017-00902-5 PMC5430818 28432312

[26] Pereiro P Librán-Pérez M Figueras A Novoa B. Conserved Function of Zebrafish (Danio Rerio) Gdf15 as a Sepsis Tolerance Mediator. Dev Comp Immunol (2020) 109:103698. 10.1016/j.dci.2020.103698 32289326

[27] Santos I Colaço HG Neves-Costa A Seixas E Velho TR Pedroso D. CXCL5-Mediated Recruitment of Neutrophils Into the Peritoneal Cavity of Gdf15-Deficient Mice Protects Against Abdominal Sepsis. Proc Natl Acad Sci USA (2020) 117:12281–87. 10.1073/pnas.1918508117 PMC7275717 32424099

[28] Wiik J Nilsson S Kärrberg C Strander B Jacobsson B Sengpiel V. Associations of Treated and Untreated Human Papillomavirus Infection With Preterm Delivery and Neonatal Mortality: A Swedish Population-Based Study. PloS Med (2021) 18:e1003641. 10.1371/journal.pmed.1003641 33970907 PMC8143418

[29] Kempf T Zarbock A Widera C Butz S Stadtmann A Rossaint J. GDF-15 is an Inhibitor of Leukocyte Integrin Activation Required for Survival After Myocardial Infarction in Mice. Nat Med (2011) 17:581–8. 10.1038/nm.2354 21516086

[30] Zhou XJ Zhang X Zhang J Zhou L Zhou TT Zhang JW. Diagnostic Value of Growth Differentiation Factor-15 and β2-Microglobulin in Children With Congenital Heart Disease Combined With Chronic Heart Failure and its Relationship With Cardiac Function. Eur Rev Med Pharmacol Sci (2020) 24:8096–103. 10.26355/eurrev_202008_22494 32767337

[31] Sarkar S Legere S Haidl I Marshall J MacLeod JB Aguiar C. Serum GDF15, a Promising Biomarker in Obese Patients Undergoing Heart Surgery. Front Cardiovasc Med (2020) 24:103. 10.3389/fcvm.2020.00103 PMC7327098 32671100

[32] Kuster N Huet F Dupuy AM Akodad M Battistella P Agullo A. Multimarker Approach Including CRP, Sst2 and GDF-15 for Prognostic Stratification in Stable Heart Failure. ESC Heart Fail (2020) 7:2230–39. 10.1002/ehf2.12680 PMC7524044 32649062

[33] Echouffo-Tcheugui JB Daya N Matsushita K Wang D Ndumele CE Al Rifai M. Growth Differentiation Factor (GDF)-15 and Cardiometabolic Outcomes Among Older Adults: The Atherosclerosis Risk in Communities Study. Clin Chem (2021) 67:653–61. 10.1093/clinchem/hvaa332 PMC8011530 33582779

[34] Sanders-van Wijk S Tromp J Beussink-Nelson L Hage C Svedlund S Saraste A. Proteomic Evaluation of the Comorbidity-Inflammation Paradigm in Heart Failure With Preserved Ejection Fraction: Results From the PROMIS-HFpEF Study. Circulation (2020) 142:2029–44. 10.1161/CIRCULATIONAHA.120.045810 PMC7686082 33034202

[35] Duong Van Huyen JP Cheval L Bloch-Faure M Belair MF Heudes D Bruneval P. GDF15 Triggers Homeostatic Proliferation of Acid-Secreting Collecting Duct Cells. J Am Soc Nephrol (2008) 19:1965–74. 10.1681/ASN.2007070781 PMC2551573 18650486

[36] Guenancia C Kahli A Laurent G Hachet O Malapert G Grosjean S. Pre-Operative Growth Differentiation Factor 15 as a Novel Biomarker of Acute Kidney Injury After Cardiac Bypass Surgery. Int J Cardiol (2015) 197:66–71. 10.1016/j.ijcard.2015.06.012 26113476

[37] Kahli A Guenancia C Zeller M Grosjean S Stamboul K Rochette L. Growth Differentiation Factor-15 (GDF-15) Levels are Associated With Cardiac and Renal Injury in Patients Undergoing Coronary Artery Bypass Grafting With Cardiopulmonary Bypass. PloS One (2014) 9:e105759. 10.1371/journal.pone.0105759 25171167 PMC4149498

[38] Cos Gomez M Benito Hernandez A Garcia Unzueta MT Mazon Ruiz J Lopez Del Moral Cuesta C Perez Canga JL. Growth Differentiation Factor 15: A Biomarker With High Clinical Potential in the Evaluation of Kidney Transplant Candidates. J Clin Med (2020) 9:4112. 10.3390/jcm9124112 PMC7766056 33419237

[39] Karakike E Kyriazopoulou E Tsangaris I Routsi C Vincent JL Giamarellos-Bourboulis EJ. The Early Change of SOFA Score as a Prognostic Marker of 28-Day Sepsis Mortality: Analysis Through a Derivation and a Validation Cohort. Crit Care (2019) 23:387. 10.1186/s13054-019-2665-5 31783881 PMC6884794

[40] Zhang Z Xu X Tian W Jiang R Lu Y Sun Q. ARRB1 Inhibits Non-Alcoholic Steatohepatitis Progression by Promoting GDF15 Maturation. J Hepatol (2020) 72:976–89. 10.1016/j.jhep.2019.12.004 31857195

[41] Liu J Kumar S Heinzel A Gao M Guo J Alvarado GF. Renoprotective and Immunomodulatory Effects of GDF15 Following AKI Invoked by Ischemia-Reperfusion Injury. J Am Soc Nephrol (2020) 31:701–15. 10.1681/ASN.2019090876 PMC7191932 32034106

[42] Kimmoun A Cotter G Davison B Takagi K Addad F Celutkiene J. Safety, Tolerability and Efficacy of Rapid Optimization, Helped by NT-proBNP and GDF-15, of Heart Failure Therapies (STRONG-HF): Rationale and Design for a Multicentre, Randomized, Parallel-Group Study. Eur J Heart Fail (2019) 21:1459–67. 10.1002/ejhf.1575 31423712

[43] Desmedt S Desmedt V De Vos L Delanghe JR Speeckaert R Speeckaert MM. Growth Differentiation Factor 15: A Novel Biomarker With High Clinical Potential. Crit Rev Clin Lab Sci (2019) 56:333–50. 10.1080/10408363.2019.1615034 31076013

[44] Wu R Wang N Comish PB Tang D Kang R. Inflammasome-Dependent Coagulation Activation in Sepsis. Front Immunol (2021) 16:641750. 10.3389/fimmu.2021.641750 PMC8007875 33796108

[45] Corre J Hébraud B Bourin P. Concise Review: Growth Differentiation Factor 15 in Pathology: A Clinical Role? Stem Cells Transl Med (2013) 2:946–52. 10.5966/sctm.2013-0055 PMC3841089 24191265

[46] Li S Li Q Zhu Y Hu W. GDF15 Induced by Compressive Force Contributes to Osteoclast Differentiation in Human Periodontal Ligament Cells. Exp Cell Res (2020) 387:111745. 10.1016/j.yexcr.2019.111745 31765611

[47] Jin Z Zhu Z Liu S Hou Y Tang M Zhu P. TRIM59 Protects Mice From Sepsis by Regulating Inflammation and Phagocytosis in Macrophages. Front Immunol (2020) 18:263. 10.3389/fimmu.2020.00263 PMC7041419 32133014

[48] Han X Li W Li P Zheng Z Lin B Zhou B. Stimulation of α7 Nicotinic Acetylcholine Receptor by Nicotine Suppresses Decidual M1 Macrophage Polarization Against Inflammation in Lipopolysaccharide-Induced Preeclampsia-Like Mouse Model. Front Immunol (2021) 12:642071. 10.3389/fimmu.2021.642071 33995360 PMC8113862

[49] Hortová-Kohoutková M Tidu F De Zuani M Šrámek V Helán M Frič J. Phagocytosis-Inflammation Crosstalk in Sepsis: New Avenues for Therapeutic Intervention. Shock (2020) 54:606–14. 10.1097/SHK.0000000000001541 PMC7566305 32516170

[50] Zhou Z Li W Song Y Wang L Zhang K Yang J. Growth Differentiation Factor-15 Suppresses Maturation and Function of Dendritic Cells and Inhibits Tumor-Specific Immune Response. PloS One (2013) 8:e78618. 10.1371/journal.pone.0078618 24236027 PMC3827235

[51] Clere-Jehl R Mariotte A Meziani F Bahram S Georgel P Helms J. JAK-STAT Targeting Offers Novel Therapeutic Opportunities in Sepsis. Trends Mol Med (2020) 26:987–1002. 10.1016/j.molmed.2020.06.007 32631717

[52] Chang X Hu LF Ma XJ Yin J Liu XY Li JB. Influence of Roflumilast on Sepsis Mice Through the JAK/STAT Signaling Pathway. Eur Rev Med Pharmacol Sci (2019) 23:1335–41. 10.26355/eurrev_201902_17028 30779101

[53] Wu J Yan X Jin G. Ulinastatin Protects Rats From Sepsis-Induced Acute Lung Injury by Suppressing the JAK-STAT3 Pathway. J Cell Biochem (2018) 22:2554–9. 10.1002/jcb.27550 30242880

[54] Beckman JD Abdullah F Chen C Kirchner R Rivera-Rodriguez D Kiser ZM. Endothelial TLR4 Expression Mediates Vaso-Occlusive Crisis in Sickle Cell Disease. Front Immunol (2021) 11:613278. 10.3389/fimmu.2020.613278 33542720 PMC7851052

