# Supplementary material for: The Clinical Value of GDF15 and Its Prospective Mechanism in Sepsis
Source: Front Immunol. 2021 Sep 8;12:710977. doi: 10.3389/fimmu.2021.710977 (PMC8456026; doi:10.3389/fimmu.2021.710977)
Supplement: Supplementary file 1 [file Table_1.doc]

| **Table S1 The sequences of primers used in Real-time PCR analysis** | | | |
| --- | --- | --- | --- |
| Species | Gene | Primer | Sequence (5' - 3') |
| Human | GAPDH | FW | CGGATTTGGTCGTATTGG |
|  |  | RV | GATGATGACCCTTTTGGC |
|  | CD80 | FW | TCTGACGAGGGCACATAC |
|  |  | RV | GGTGAGGCTCTGGAAAAC |
|  | CD163 | FW | AGACTGTTAGGGAAGGTGT |
|  |  | RV | GTGTTTGTTGCCTGGATT |
| Mouse | GAPDH | FW | AACGGATTTGGCCGTATTGG |
|  |  | RV | CATTCTCGGCCTTGACTGTG |
|  | CD80 | FW | GCCTTGCCGTTACAACTCT |
|  |  | RV | GAAACTGGTGCAGGAGGAT |
|  | CD163 | FW | CCTCTGCTGTCACTAACGC |
|  |  | RV | CTGCCAGACGAATATCTATGT |
